# Zygomatic implant penetration to the central portion of orbit: a case report

**DOI:** 10.1186/s12886-021-01846-1

**Published:** 2021-03-06

**Authors:** Luan Mavriqi, Felice Lorusso, Roberto Conte, Biagio Rapone, Antonio Scarano

**Affiliations:** 1 University of Albania University, Tirana, Albania; 2 Department of Innovative Technologies in Medicine & Dentistry, University of Chieti-Pescara, Chieti, Italy; 3 Department of Basic Medical Sciences, Neurosciences and Sense Organs, “Aldo Moro” University of Bari, 70121 Bari, Italy; 4 Department of Medical, Oral and Biotechnological Sciences and CAST, University G. D’Annunzio of Chieti-Pescara, Via dei Vestini, 31-66100 Chieti, CH Italy

**Keywords:** Zygomatic implants, Maxillary atrophy, Fixed prosthesis, Case report

## Abstract

**Background:**

Zygomatic implants have been proposed in literature for atrophic maxillary fixed oral rehabilitations. The aim of the present research was to evaluate, by a clinical and tomography assessment, a surgical complication of a zygomatic implant penetration to the orbit.

**Case presentation:**

A 56 year-old female patient was visited for pain and swelling in the left orbit after a zygomatic implant protocol. The orbit invasion of the zygomatic implant screw was confirmed by the CBCT scan. The patient was treated for surgical implant removal and the peri- and post-operative symptoms were assessed. No neurological complications were reported at the follow-up. The ocular motility and the visual acuity were well maintained. No purulent secretion or inflammatory evidence were reported in the post-operative healing phases.

**Conclusion:**

The penetration of the orbit during a zygomatic implant positioning is a surgical complication that could compromise the sight and movements of the eye. In the present case report, a zygomatic implant removal resulted in an uneventful healing phase with recovery of the eye functions.

## Background

Dental implants are extensively used in clinical practice for replacing missing teeth and as an adjunct in the reconstruction of jaws and teeth. Severe maxillary atrophy can be observed in patients with an edentulous state. In fact, dental extractions induce bone loss and alveolar bone leading to expansion of the maxilla sinuses, to severe atrophy and to a decrease in volume, making it difficult to insert dental implants. So the rehabilitation of severe maxillary atrophies related to the oncologic resection, traumatic loss or the loss of dental elements, requires treatment with bone regeneration or reconstructive surgical procedures, preparatory to implant-prosthetic support [1]. Different techniques are used in clinical practice to increase hard and soft tissue, usually autologous bone is used, such as the use of bone intraoral and / or extraoral grafts with or without membranes [2], sometimes associated with Type I Le Fort osteotomies [3]. A non-invasive technique involves the use of completely removable dentures, however this solution may not meet the psychological, functional and social needs of the patient. Another technique, such as zygomatic fixed implant rehabilitation could represent a treatment option for severe partial or complete atrophy of the maxillary. The zygomatic dental implant has proved to be an effective option for the fixed rehabilitation of maxillary atrophic edentulous ridges, as well as defects in the maxillectomia [4]. In this case one or two long implants can be positioned that involves the insertion of implants through the sinus intra route and guided insertion through the execution of a lateral trapdoor bone, without lifting the Schneider membrane [5]. The original technique proposed by Branemark has been modified to provide for the preservation and lifting of the Schneiderian membrane, contextual to the procedure. Stella and Warner have proposed a variant of the zygomatic implant placement technique (sinus slot technique), which does not require detachment of the Schneider membrane [6]. Regarding this, a further technical variant with extrasinus approach has been proposed, whose implant route is completely external to the cavity of the maxillary sinus. Several retrospective studies document a percentage of zigomatic implant survival rate of 90–100% [7–9].

In the literature different geometries and implant designs have been proposed in order to facilitate an optimal positioning of the fixture and a long-term maintenance of osseointegration of the placed implants [10–12].

In this case report, we present a case of invasion of the orbital cavity after placement of a zygomatic denture-anchoring implant and it is described in line with the SCARE criteria [13].

## Case presentation

**Fig. 1:**
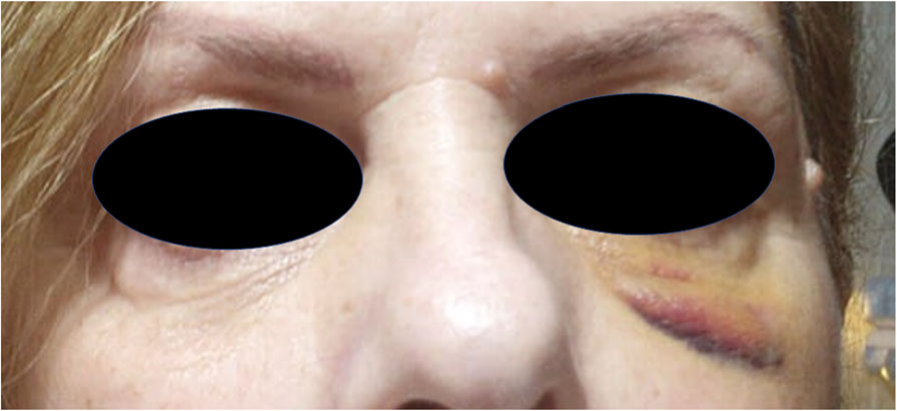
CT image after surgery showing the zygomatic implant tip in the lateral orbit

**Fig. 2:**
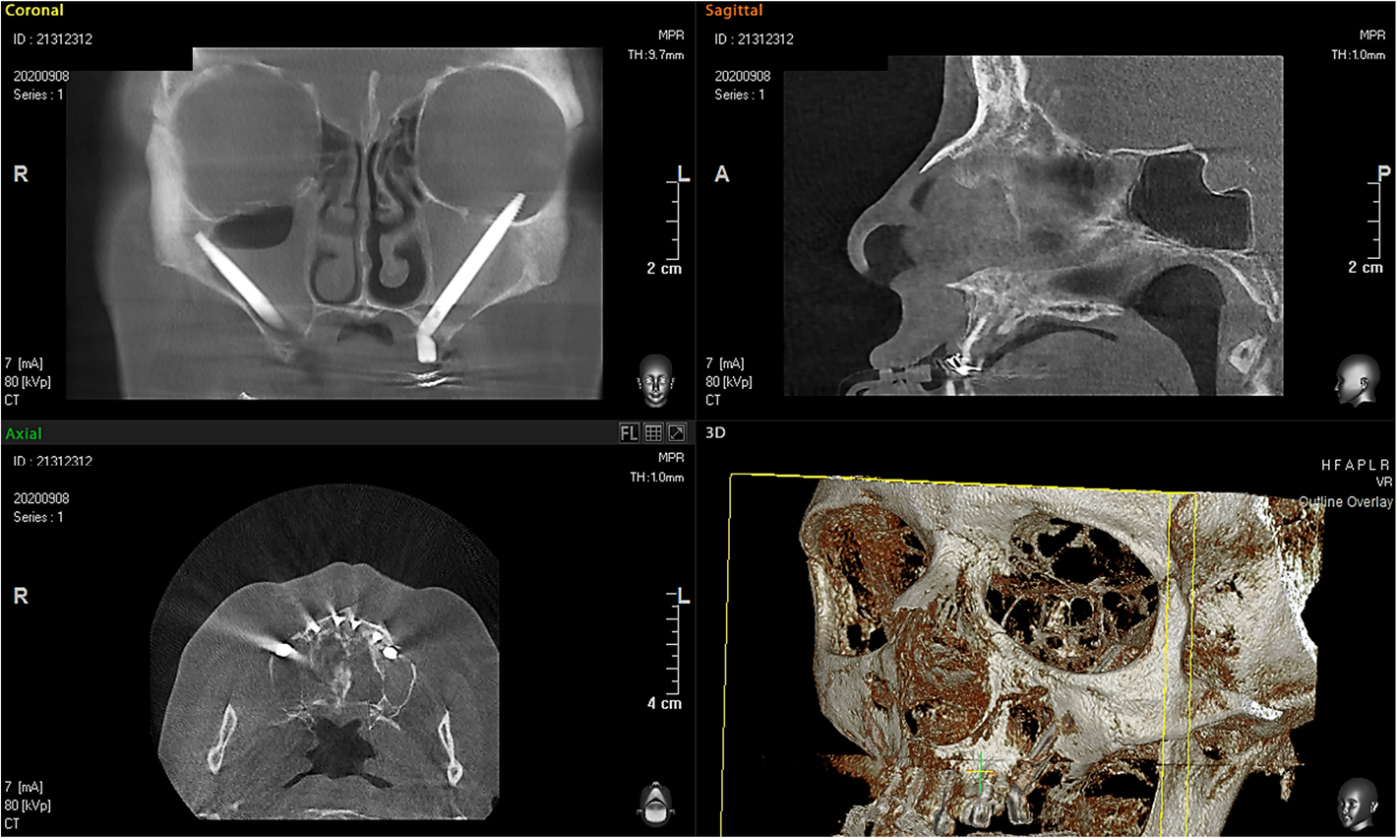
Placement of two zygomatic implants

**Fig. 3:**
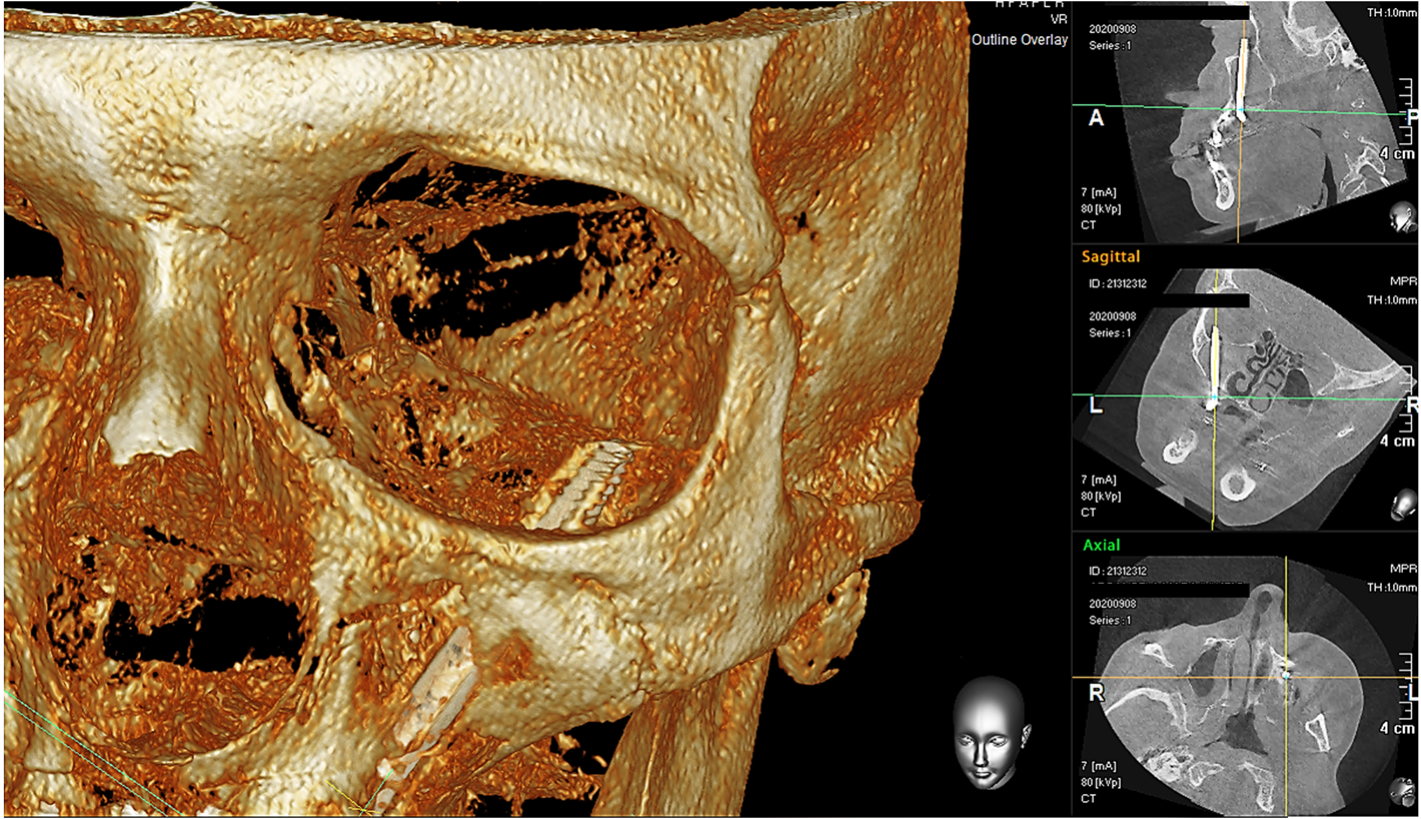
The left zygomatic implant has an intra-sinusal course and involved the left orbit

The present study was conducted according the ethical principles of the Declaration of Helsinki amended in 2013. A 56-year-old woman was seen in our Outpatient Clinic for pain and swelling in her left eye, which developed following a zygomatic implant (Fig. 1). Different systemic disorders such as blood diseases, viral and bacterial infections, multiple sclerosis, sarcoidosis, drug-induced diseases, metastasis, and local pathologies such as jaw bone fractures, third molar extractions, neoplasms, and metastasis of cancer, which all have been proven to be responsible for such symptoms, were excluded. After 1 day without any changes in symptomatology, the patient was referred to the Department of Oral Surgery of the University of Chieti-Pescara accompanied by her oral surgeon and submitted written informed consent. The procedure had apparently involved placement of bilateral zygomatic implants. No other specific details of the implant surgery procedure were available. After the zygomatic implant the patient developed pain in the region of the left orbit, persistent anaesthesia, diplopia and difficulty in moving the eye. Examination by three-dimensional X-ray (CBCT) demonstrated a zygomatic implant penetrating the central part of the left orbit (Figs.2, 3). The muscles and their insertion appeared intact and odontogenic sinusitis was observed. An ophthalmological consultation was requested, and it was decided to remove the implant. The zygomatic implant was removed from the orbit, through the mouth with a loosening movement. Local anesthesia was administered with Articaine® (Ubistesin 4%—Espe Dental AG, Seefeld, Germany) associated with epinephrine 1:100.000. Full-thickness flaps were elevated to expose the alveolar crest and zygomatic implant in the left maxilla. No bleeding was seen, and no orbital intervention was needed. A systemic antibiotic for 5 days with amoxicillin 1 g and betamethasone 1 mg for 6 days was prescribed. In order to preserve the intestinal microbiome, the intake of lactic acid bacteria (Biocult Strong, Italfarmacia, Rome-Italy) was also prescribed. The flap was sutured with non-absorbable thread 4.0 (Assut Europe, Magliano dei Marsi, AQ) leaving the distal outlet free to facilitate inflammatory exudate drainage in the first hours after surgery [12]. Seven days after the procedure the surgical suture was removed. No other ocular treatment was needed, and no bleeding or congiuntival irritation was seen. Motility of the right eye was normal. After 1 week no pain in the region of the left orbit, persistent anaesthesia, diplopia nor difficulty in moving the eye were detected. After 2 weeks from the surgical intervention the patient reported sensitivity of the area, but no case of neurological damage was observed. No other clinical signs were present after 4 weeks. No pain or purulent secretion on palpation was reported to be associated with any of the inserted implants.

## Discussions and conclusions

Today there is a great interest in zygomatic implant techniques because they offer an additional treatment option for rehabilitation of patients with an extremely atrophic upper jaw and can potentially also improve facial soft tissues. This technique is a more invasive procedure, but it may be the best or only option in cases of extreme atrophy and where bone reconstructive options are contra-indicated, failed [14] or unwanted by the patient [15, 16]. At patient was proposed a sinus lifting and vertical bone regeneration solution but the probability of success both solution were very low, so the patient accepted the placement zygomatic implants. Some authors reported zygomatic implants being associated with serious types of complications, such as buccos-inusal fistula persistent, maxillary sinusitis, dehiscence around the implants [17], and infraorbital nerve damage [18]. Also accidental implant intracerebral penetration or even penetration into the nasal cavity have been described [19, 20]. Accidental implant penetration into the orbit is one of the most serious complications following zygomatic placement [21]. In our case report we have shown that the patient demonstrated physiological abduction and elevation of the right eye although damage to the extraocular muscles was excluded. Krauthammer et al. reported a clinical case with damage to the lateral rectus and the inferior oblique muscles with restricted abduction and elevation of the right eye [21]. After one-month, scleral adhesions were observed in the temporal portion of the rectus muscles, with restriction on abduction and elevation of the damaged eye during forced duction test. A surgical intervention to the tendon transpositions of the superior and inferior rectus muscles was performed, in an attempt to compensate the diplopia [22].

Also, orbital floor fracture has been reported during zygomatic implant placement [23]. This complication can produce severe right periorbital swelling, conjunctival hematoma, extraocular muscle injury that causes diplopia upon manual lid elevation and inability to open the eye.

When there is invasion of the orbital cavity, fibrosis or pushing of the bone fragment into the orbital cavity can be determined, so the displacement of the extraocular muscles determines diplopia and reduction in movement of the eye. Hematoma and fibrosis surrounding the inferior oblique muscle may disappear after 1 month, in some cases an extensive and persistent fibrosis surrounding the inferior oblique muscle is possible.

If these symptoms persist it is necessary to repair the orbital floor fracture by implanting a high-density porous polyethylene implant. The integrity of the bulb and its movements are also important from a neurological point of view for the entire visual process, and it can be useful to evaluate the motion of the tear film, detectable en-face through optical coherence tomography [24].

The placement of zygomatic implants (ZI) should be made only by surgeons with extensive experience, to reduce post-operative complications. In fact, different anatomical structures adjacent to the zygomatic bone, such as the orbit, can be damaged during zygomatic implant placement [21]. Severe pain of the eye and diffused swelling of the upper and lower eyelid were reported by Van Camp et al. [25].

The authors performed an active drainage of blood from within the orbit with Penrose drains and prescription of methylprednisolone 40 mg (four times a day) and acetazolamide 500 mg. After many months, it is possible to observe in some cases infection or cutaneous fistula in the zygomatic-orbital area. The use of two or three zygomatic implants on each side increases these complications because the height and thickness of the zygomatic bone are small and distance between implant and orbit is very short [26].

Takamaru et al. [26] measured thickness and height of the zygomatic bone process and proposed a novel method for zygomatic implant insertion that is safer than existing methods. The apex zygomatic implant insertion point should be positioned infero-anterior to the 90° angle point to take advantage of the greater bone thickness and major distance from orbit and infraorbital nerve. An important anatomical landmark during ZI is the infraorbital nerve, the drill should be positioned lateral to the nerve, toward the superior portion of the zygomatic bone that composes the lateral orbital rim.

These anatomical considerations of the patient are important during zygomatic implant placement. So, it is prudent to modify the implant angulation based on anatomic patient considerations. Computed tomography is important for evaluation of the zygomatic implant site and the sinus status, as well as for the implant path. In fact, cone beam computed tomography is crucial to determine the amount of bone in the zygomatic arch and in the residual alveolar crest which have to be explored in both horizontal and vertical dimensions. It is important to take a stereolithography model realized by the DICOM files and a 3D printer, that is a good aid for this type of surgery as it faithfully reproduces the bony anatomy of the patient, allowing to assess the exact size and position of the implants. In this case report, the surgeon who placed the implants used the intranasinus technique. This technique increases the risk of orbit invasion, because there is no vision and control of the drills during implant bed preparation. To avoid this complication a concept called the zygomatic anatomy-guided approach (ZAGA) [27] was proposed; a modification of the original zygomatic implant technique that focuses on interindividual anatomic differences. For this reason, the invasion of the orbit during zygomatic surgery described in this case report is to be considered not just a complication but as a case of medical malpractice. The planning of zygomatic implant is “a must” for the surgeon who performs this type of intervention: proper planning is essential for the success of any surgery. This represents the greatest lesson that can be learned from the described case.

In conclusion, the placement of zygomatic implants should be made only by surgeons with extensive experience, to reduce the post-operative complications, especially in a period in which there is an increase in the use of zygomatic implants.

However the zygomatic implant technique represents a noble alternative to regenerative bone procedures taking advantage of the available bone, anchored in the zygomatic region to native and non-regenerated bone, with obvious biomechanical advantages [2, 8].

## Data Availability

Not applicable.
